# Clinical efficacy and safety of tigecycline based on therapeutic drug monitoring for carbapenem-resistant Gram-negative bacterium pneumonia in intensive care units

**DOI:** 10.1186/s12879-023-08815-7

**Published:** 2023-11-27

**Authors:** Xiang-rong Bai, Zhi-zhou Wang, Wen-chao Li, Yan-gai Wang, Ran Lou, Xin Qu, Linlin Fan, Wei Zhang, Yan-chuan Wu, Su-ying Yan, Lan Zhang

**Affiliations:** 1 Department of Pharmacy, Xuan Wu Hospital Capital Medical University, National Gerontic Disease Clinical Research Center, No. 45 Changchun Street, Xi Cheng District, Beijing, 100053 China; 2 Department of Intensive Medicine, Xuan Wu Hospital Capital Medical University, National Gerontic Disease Clinical Research Center, Beijing, 100053 China; 3 Intensive Care Unit, Department of Neurosurgery, Xuan Wu Hospital Capital Medical University, National Gerontic Disease Clinical Research Center, Beijing, 100053 China; 4 Neurology Intensive Care Unit, Xuan Wu Hospital Capital Medical University, National Gerontic Disease Clinical Research Center, Beijing, 100053 China; 5 Department of Pulmonary and Critical Care Medicine, Xuanwu Hospital Capital Medical University, Beijing, 100053 China; 6 Central Laboratory of Xuanwu Hospital, Capital Medical University, Beijing, 100053 China

**Keywords:** Tigecycline, Therapeutic drug monitoring, Multidrug-resistant Gram-negative bacteria, Fibrinogen decline, Pneumonia

## Abstract

**Background:**

We investigated the associations between the different doses of tigecycline, its efficacy and safety, and the role of tigecycline therapeutic drug monitoring for patients in the intensive care unit.

**Methods:**

This study was a single-center cohort including patients infected with multidrug-resistant *Acinetobacter baumannii* (MDR-AB) and multidrug-resistant *Klebsiella pneumoniae* (MDR-KP) causing pulmonary infections. The steady-state plasma concentration after tigecycline administration was determined by High-Performance Liquid Chromatography (HPLC) in patients admitted to the ICU between October 2020 and December 2021. Multivariate analyses of tigecycline’s clinical efficacy and safety were performed to control confounding factors.

**Results:**

For this study, we included 45 patients and 45 blood samples to determine steady-state trough concentrations of tigecycline. All patients were divided into the High Dose (HD) and Standard Dose (SD) groups. The median trough concentration of tigecycline was 0.56 μg/mL in the HD group, which was higher than in the SD group (0,21 μg/mL), *p* = 0.000. There was no significant difference between the two groups of patients in terms of bacterial eradication rate, mortality rate, and clinical efficacy. Multiple regression analysis showed that the ICU days were correlated with mortality OR 1.030(1.005–1.056), *p* = 0.017. APACHE II was significantly associated with clinical efficacy OR 0.870(0.755–1.002), *p* = 0.045. The level of fibrinogen decline in the HD group was significantly higher than in the SD group (-3.05 ± 1.67 vs -1.75 ± 1.90), *p* = 0.038. We identified that age and tigecycline treatment duration influenced fibrinogen decline.

**Conclusions:**

Tigecycline plasma concentrations are significantly increased when using a high dose. However, the plasma concentration of tigecycline is not correlated with clinical efficacy and adverse reactions. Fibrinogen decline appears to be related to the patient’s age and days of tigecycline. Large sample data are still needed to confirm the clinical guidance significance of tigecycline TDM.

**Supplementary Information:**

The online version contains supplementary material available at 10.1186/s12879-023-08815-7.

## Background

The incidence of pneumonia caused by Gram-negative organisms has significantly increased in the ICU [1]. Multidrug-resistant (MDR) Gram-negative bacteria, especially *Pseudomonas aeruginosa*, *Acinetobacter baumannii,* and Enterobacteriaceae members, are predominant. Multidrug-resistant *A. baumannii*((MDR-AB) and Carbapenemase-producing *Klebsiella pneumoniae* (KPC-KP) infections are significant causes of mortality in intensive care units [2]. Managing these infections is difficult due to their antibiotic resistance, particularly to carbapenem antibiotics. Tigecycline is one of the few broad-spectrum antibiotics highly active in treating pneumonia caused by MDR bacteria,including MDR-AB and KPC-KP [3]. It is off-label used in ventilator-associated pneumonia hospital-acquired pneumonia (HAP) caused by MDR pathogens, especially carbapenem-resistant (CR) bacteria [4]. The recommended initial dose of tigecycline for injection is 100 mg, followed by 50 mg every 12 h. A meta-analysis study indicated that higher doses of tigecycline lead to lower all-cause mortality and higher clinical cure and microbiological eradication rates [5]. Nowadays, the IDSA guidelines recommend high-dose tigecycline (initial dose of 200 mg, followed by 100 mg every 12 h) as an alternative option [6]. However, it will increase the risk of adverse effects of tigecycline for critically ill patients. Gastrointestinal symptoms, including nausea, vomiting, and diarrhea, are the most common adverse reactions in all-case post-marketing surveillance studies [7]. Some studies showed that high-dose tigecycline treatment in ICU patients may decrease plasma fibrinogen levels and activate partial thromboplastin time (APTT) values [8–10]. Coagulopathy has been reported as a side effect of high-dose tigecycline in the FDA adverse event reporting systems [10]. A study indicated that age, days of medication, and baseline fibrinogen levels were associated with hypofibrinogenemia [11]. Therefore, preventing or detecting coagulation abnormalities in time is crucial, especially for ICU patients.

Therapeutic drug monitoring (TDM) of antibiotics was recommended for critically ill patients to optimize drug effectiveness and adjust the dosage [12]. A study found that trough tigecycline concentrations can predict liver toxicity [13]. However, there is limited clinical experience with tigecycline TDM, and no evidence exists to determine whether tigecycline TDM can predict the risk of hypofibrinogenemia. Therefore, we evaluate the clinical efficacy by monitoring the plasma concentration of tigecycline and provide a reference for early prediction of coagulation abnormalities.

## Material and methods

### Study design and participants

This retrospective cohort study was conducted in a single health system from December 2020 to October 2021 at a tertiary-care teaching hospital in China. The study included patients over 18, diagnosed with pulmonary infection, and treated with tigecycline. Blood samples were collected from the patients had to fulfill the following mandatory criteria: samples were taken from patients who had received tigecycline therapy for > 72 h (2) adult patients (> 18 years) who needed treatment with tigecycline which bronchoalveolar lavage fluid culture was MDR-AB and KPC-KP. Respiratory tract secretion test was performed daily. Exclusion criteria were pregnancy, (2) liver or kidney failure, (3) non-compliance with the dosing regimen, and (4) infection in other sites. All patients were divided into two groups according to the medical orders by the physician: High Dose (HD) (loading dose 200 mg, maintenance dose 100 mg q12h) group and Standard Dose (SD) (loading dose 100 mg, maintenance dose 50 mg q12h) group, based on their tigecycline dosage. The follow-up time for all patients was transition from the ICU or discharge. Hospital-acquired pneumonia was diagnosed based on IDSA guidelines [14].

### Blood sampling and analytical assays

To obtain total tigecycline concentration samples for analysis, 0.5–1.0 mL of peripheral vein blood was collected from patients at trough concentration points or other times after administration. The collected blood was placed in vacutainer tubes without anticoagulant and then centrifuged at 3,000 rpm for 10 min at 10◦C, and the serum was collected and stored at −40℃ until analysis. An HPLC method was used to determine the concentration of tigecycline in the serum, with a calibration curve ranging from 0.10–5.00 μg/mL showing good linearity. The intraday and interday precision RSD for low, medium, and high-dose quality control samples were less than 10%, while the extraction recovery ranged from 99.43% to 116.68% [15].

### Data collection

Patient demographic characteristics, including gender, age, Charlson Comorbidity Index, mechanical ventilation, catheter indwelling, and steroid use during treatment, were collected. Data on the usage, dosage, course of treatment, and combination of tigecycline were also collected. Changes in body temperature (T), respiration (R), pulse (P), chest imaging, and laboratory indexes were collected before and after treatment. Laboratory tests, including White blood cell count (WBC), Albumin (ALB), platelet(PLT), procalcitonin (PCT), C-reactive protein (CRP), Albumin, Fibrinogen level(Fib), Prothrombin time (PT), and Activated partial prothrombin time (APTT), were also collected before tigecycline initiation and within 1 week after discontinuation.

### Microbiological analysis

All the MDR-KP and MDR-AB were isolated for evaluation with 13 different antibiotics using the VITEK-2 compact system (bioMérieux, France). The minimum inhibitory concentration (MIC) was routinely determined by Clinical and Laboratory Standards Institute (CLSI) guidelines. The results for CR *A. baumannii* and CRKP were interpreted as susceptible (S), intermediate (I), or resistant (R), based on the breakpoints for Enterobacteriaceae strains released by the US Food and Drug Administration (FDA) (S ≤ 2, I = 4, and R ≥ 8 mg/L for minimum inhibitory concentration (MIC) testing by using the E-test method (BIO-KONT, Wenzhou, China) [16].

### Clinical outcomes

#### All-cause death in the ICU

The patient died during the follow-up period.

Clinical efficacy was divided into effective clinical treatment (improvement of body temperature, symptoms and signs, and improvement or no progress of pulmonary imaging) and ineffective clinical treatment (lack of improvement or aggravation in body temperature and vital signs, progress of pulmonary imaging or death of patients due to infection).

#### Eradication of pathogens

The microbiology efficacy was divided into eradication (negative bacterial culture results), hypothetical eradication (clinically effective but not confirmed due to the inability to obtain respiratory specimens), and replacement (MDR-AB and KPC-KP were clear, while other bacteria were detected) and re-infection (MDR-AB and KPC-KP were still detected in the culture). The microbiology eradication rate = (number of cleared cases + assumed number of cleared cases + number of replacement cases)/total number of cases × 100% [17]. The duplicated cases (multiple isolates in the same patient) were not included.

### Safety

#### Changed of coagulation values

The changes values of platelet (PLT), fibrinogen level (Fib), prothrombin time (PT), and activated partial prothrombin time (APTT) were collected before and after the administration of tigecycline.

#### The decline of fibrinogen

< 1.0 ~ 0.75 times the lower limit of normal value; If the baseline value is abnormal, it is lower than the baseline < 25% according to the Common Terminology Criteria for Adverse Event (CTCAE) v.5.0 guidelines [18].

Two independent investigators performed confirmation of clinical and safety outcomes.

### Statistical analysis

Continuous variables were described using mean and standard deviation or median with interquartile range, while categorical variables were described using frequency or percentage. The Student t-test, or a nonparametric test, was used to compare continuous variables, and the X^2^ test, or Fisher’s exact test, was used to compare categorical variables. Covariates with *p* < 0.05 in the univariate analysis were included in the multivariate analysis. IBM SPSS Statistics (version 25.0, IBM, Armonk, NY) was used for statistical analyses, and a two-sided *p* < 0.05 was considered statistically significant. All graphs were created using GraphPad Prism (version 9.0, GraphPad Software, La Jolla, CA).

## Results

### Baseline demographics

Between October 2020 and December 2021, we identified 139 patients who met the inclusion criteria. After applying the exclusion criteria, 45 patients were included in the study, with 13 in the high-dose group and 32 in the standard-dose group. See the flow chart in the Supplementary Fig. S1. Baseline characteristics of the two groups are presented in Table 1, and all patients were consistent at baseline. The media concentrations of tigecycline differed significantly between the two groups (0.56 μg/mL vs 0.21 μg/mL). The distribution of tigecycline plasma concentration in two groups of patients is presented in the Supplementary Fig. S2. All 45 patients were treated with a combination of tigecycline regimens, with 27 patients being carbapenem-resistant *A. baumannii* and 18 patients being carbapenem-resistant* K. pneumoniae*, which were sensitive to tigecycline. However, the two groups had no significant difference in bacterial eradication, mortality, or clinical efficacy.

**Table 1:** Baseline demographics

Demographic	HD group *N* = 13	SD group *N* = 32	*P*-value
Gender, male/female	11/2	25/7	0.622
Age (yr)	62.09 ± 15.57	63.62 ± 15.79	0.770
BMI	25.24 ± 3.94	25.12 ± 6.52	0.951
CCI median (IQR)	1 (0.5–3)	1 (0.0–2.75)	0.219
APACHE II	20.94 ± 5.72	19.91 ± 5.58	0.659
CPIS median (IQR)	8 (6.5–8.5)	7 (7–8)	0.614
SOFA median (IQR)	5 (3–5.5)	3 (2–5)	0.673
Mechanical ventilation	11	25	1.000
Duration of tigecycline treatment (d)	12.31 ± 5.63	16.63 ± 10.02	0.152
Through concentration (μg/mL), median (IQR) mean	0.56 (0.43–0.81)	0.21 (0.15–0.36)	0.000
ICU days median (IQR)	38 (25–63)	40 (22.5–59)	0.981
Bacterial eradication	2	3	0.617
Mortality	2	9	0.467
Clinical efficacy	12	20	0.070
Combination with tigecycline			
Carbapenems	4	16	0.336
Aminoglycosides	3	12	0.492
Polymyxin B	4	0	-
Cefoperazone sulbactam	2	3	0.617
Ceftadine	0	1	-

*HAP* Hospital-acquired pneumonia, *CAP* Community-acquired pneumonia, *BMI* Body Mass Index, *CCI* Charlson Comorbidity Index, *APACHE II* Acute Physiology and Chronic Health Disease Classification System II, *SOFA* Sequential Organ Failure Assessment, *CPIS* The Clinical Pulmonary Infection Score

### Antimicrobial susceptibility test

All isolates were resistant to IPM (Imipenem), MEM (meropenem), AMP (ampicillin), CAZ: (ceftazidime), CTX (cefotaxime), CRO (ceftriaxone), PIP (piperacillin), TZP (piperacillin and tazobactam), MNO (minocycline), and ATM (aztreonam) except CT (colistin) and AMK (amikacin). Thirty-three isolates (73.3%) showed MIC = 2 mg/L, 8 (17.8%) MIC = 1 mg/L, 2 (4.4%) MIC ≤ 0.5 mg/L, 2 (4.4%) MIC = 4 mg/L which was intermediate to tigecycline. Please see Table 2. There were no significant clinical outcomes and bacterial clearance rates between the two groups for MIC = 2 mg/L. Please see the results in Supplementary Table 1.

**Table 2:** The distribution of MIC in two groups

MIC (mg/L)	HD group (*N* = 13)	SD group (*N* = 32)	*P*-value
< 0.5	2	0	0.471
1	1	7	
2	10	23	
4	0	2	

### Baseline labs

Before using tigecycline, there were no statistically significant differences in coagulation indicators such as PT, APTT, and Fib between the two groups. Similarly, no statistically significant differences existed in inflammatory response indicators such as CRP and PCT. Please refer to Table 3 for further details.

**Table 3:** Laboratory test values analyzed in both groups

Lab value	High dose *N* = 13	SD dose *N* = 32	*P*-value
WBC (10^9^/L)	9.71 ± 4.68	11.18 ± 5.28	0.302
ALB	27.73 ± 2.77	29.39 ± 3.06	0.993
Fib(g/L)	5.5 ± 1.67	6.0 ± 1.79	0.445
APTT(s)	42.43 ± 8.86	40.16 ± 6.91	0.329
PLT (10^9^/L)	269.9 ± 140.71	247.06 ± 147.37	0.861
PT(s)	13.92 ± 1.34	13.7 ± 0.81	0.084
PCT (ng/mL)	0.2 (0.09–0.46)	0.22 (0.12–0.72)	0.494
CRP (mg/L)	39 (18–104)	46 (16–67)	0.897
ALT (IU/L)	29 (19–97)	37 (16–42)	0.841
AST (IU/L)	37 (25–84)	37 (25–41)	0.520
ALP(IU/L)	71 (60–125)	80 (65–101)	0.743
TB (μmol/L)	7.35 (5.7–9.15)	9.31 (5.6–13.2)	0.340

*WBC* White blood cell, *ALB* Albumin, *Fib* Fibrinogen, *APTT* Activated partial prothrombin time, *PT* Prothrombin time, *PLT* Platelet, *PCT* Procalcitonin, *CRP* C-reactive protein, *ALT* Alanine transaminase, *AST* Aspartate aminotransferase, *ALP* Alkaline phosphatase, *TB* Total bilirubin

### Logistic regression analysis of mortality in ICU

Multivariate logistic regression analysis was performed to evaluate the mortality in the ICU (Table 4). The results indicated that ICU days were a risk factor after adjusting for these variables (OR 1.030 95% CI 1.005–1.056 *p* = 0.017).

**Table 4:** Univariate and multivariate analysis of the mortality in the ICU

Variable	Univariate analysis			Multivariate analysis		
	OR	95% CI	*p*-value	OR	95% CI	*p*-value
Age	0.986	0.912–1.067	0.732			
Gender (female)	1.417	0.137–14.626	0.770			
BMI	0.909	0.700–1.181	0.475			
CCI	1.504	0.797–2.835	0.208			
APACHE II	1.157	0.916–1.463	0.222			
CPIS	1.389	0.563–3.417	0.474			
SOFA	1.215	0.855–1.727	0.278			
Duration of tigecycline treatment	0.326	0.813–1.071	0.326			
Trough concentration	0.254	0.536–10.573	0.254			
ICU days	1.079	1.000–1.164	0.051	1.030	1.005–1.056	0.017
Mechanical ventilation	0.476	0.048–4.699	0.525			

*BMI* Body Mass Index, *CCI* Charlson Comorbidity Index, *APACHE II* Acute Physiology and Chronic Health Disease Classification System II, *SOFA* Sequential Organ Failure Assessment, *CPIS* The Clinical Pulmonary Infection Score, *TG* Tigecycline, *CI* Confidence Interval

### Logistic regression analysis of clinical efficacy

Multivariate logistic regression analysis was performed to evaluate the clinical efficacy of tigecycline (Table 5). The results showed that APACHE II was significantly associated with clinical efficacy after adjusting for potential confounding variables (OR 0.870 95% CI 0.755–1.002 *p* = 0.045).

**Table 5:** Univariate and multivariate analysis of clinical efficacy

Variable	Univariate analysis			Multivariate analysis		
	OR	95% CI	*p*-value	OR	95% CI	*p*-value
Age	0.938	0.904–1.067	0.230			
Gender (female)	0.408	0.003–0.845	0.038	0.213	0.038–1.197	0.079
BMI	1.566	0.984–2.493	0.058			
CCI	1.080	0.592–1.972	0.802			
APACHE II	0.720	0.543–0.955	0.023	0.870	0.755–1.002	0.045
CPIS	0.889	0.348–2.274	0.807			
SOFA	1.021	0.584–1.784	0.942			
Duration of TG treatment	1.195	0.965–1.480	0.102			
Trough TG concentration	1.084	0.955–18.858	0.956			
ICU days	1.030	0.955–1.113	0.460			
Mechanical ventilation	18.79	0.570–619.68	0.100			

*BMI* Body Mass Index, *CCI* Charlson Comorbidity Index, *APACHE II* Acute Physiology and Chronic Health Disease Classification System II, *SOFA* Sequential Organ Failure Assessment, *CPIS* The Clinical Pulmonary Infection Score, *TG* Tigecycline, *CI* Confidence Interval

### Safety

#### Changes in coagulation

This study revealed that the decrease in fibrinogen levels was significantly higher among high-dose patients compared to the standard-dose group, with statistical significance (*p* = 0.038). Please refer to Table 6 for further details.

**Table 6:** Changes in coagulation in two groups after tigecycline discontinuation

Lab value changed	HD group (*N* = 13)	SD group (*N* = 32)	*P*-value
ΔFib	-3.05 ± 1.67	-1.75 ± 1.90	0.038
ΔAPTT	7.25 ± 9.32	6.93 ± 9.19	0.918
ΔPT	0.95 ± 1.53	1.07 ± 1.52	0.860
ΔPLT	47.15 ± 152.01	-7 ± 144.9	0.374

*Δ* Changed, *Fib* Fibrinogen, *APTT* Activated partial prothrombin time, *PT* Prothrombin time, *PLT* Platelet

#### Logistic regression analysis of fibrinogen decline

After conducting a multivariate regression analysis on the decline of fibrinogen, the study revealed that age (OR 0.231, 95% CI 0.086–0.616, *p* = 0.003) and the duration of tigecycline treatment (OR 1.115, 95% CI 1.006–1.235, *p* = 0.038) were the primary influencing factors. However, no significant correlation was found between the trough concentration of tigecycline in the blood and the decline of fibrinogen, as demonstrated in Table 7.

**Table 7:** Univariate and Multivariate analysis of tigecycline-induced fibrinogen decline

Variable	Univariate analysis			Multivariate analysis		
	OR	95% CI	*p*-value	OR	95% CI	*p*-value
Age	0.986	0.897–1.083	0.467	0.231	0.086–0.616	0.003
BMI	1.175	0.706–1.954	0.535			
CCI	5.869	0.703–48.992	0.102			
APACHE II	1.131	0.897–1.083	0.438			
CPIS	1.122	0.427–2.951	0.815			
SOFA	0.772	0.348–1.499	0.722			
Duration of TG treatment	0.820	0.844–1.046	0.011	1.115	1.006–1.235	0.038
Trough TG concentration	5.89	0.004–8.502	0.146			
ICU days	1.013	0.941–1.090	0.730			

*BMI* Body Mass Index, *CCI* Charlson Comorbidity Index, *APACHE II* Acute Physiology and Chronic Health Disease Classification System II, *SOFA* Sequential Organ Failure Assessment, *CPIS* The Clinical Pulmonary Infection Score, *TG* Tigecycline, *CI* Confidence Interval

## Discussion

In this study, we used an established HPLC method to evaluate the concentration of tigecycline in ICU patients. All patients had consistent baselines; no significant difference was observed between the two groups. All *K. pneumoniae* isolates were resistant to ß-lactam antibiotics and carbapenems. The prevalent resistance to carbapenems was detected in all 52 isolates, whereas the presence of metallo-beta-lactamase was not detected. Meropenem-vaborbactam or ceftazidime–avibactam is not available in our hospital formulary. However, according to the European Society of Clinical Microbiology and Infectious Diseases (ESCMID) guidelines for patients with severe CRKP infections [19], tigecycline is still an option.

Our study found that patient mortality and clinical efficacy were associated with APACHE II sore and ICU days, respectively. However, the blood concentration of tigecycline was not correlated with clinical effectiveness and mortality. Regarding safety, the fibrinogen decline was not related to the blood concentration of tigecycline, except for the patient’s age and days of tigecycline. It suggests that close monitoring of the days of treatment is necessary for ICU patients.

Studies have shown that the clinical efficacy of tigecycline for MDR Gram-negative bacteria was 57.57%-71.43%, and the bacterial eradication rate was 25%-56.5% [20]. Our study also demonstrated a clinical treatment efficacy of 71%, consistent with the literature. However, the bacterial eradication rate observed in our study was 11%, which was lower than reported in previous studies.

The ICU mortality rate of 24.4% was comparable to a meta-analysis study with a mortality rate of 31.4% [21]. There is a tendency for higher mortality in the HD group with decreased fibrinogen, but it is not statistically significant. These results were also consistent with Zhang’s study, which found that hospital mortality was higher in patients with hypofibrinogenemia than those with normal fibrinogen. Nevertheless, the patients in this study were not critically ill [22].

Critically ill patients can experience a range of pathophysiological changes that complicate antibiotic dosing. Therefore, knowledge of the pharmacokinetic and pharmacodynamic properties of the antibiotics used to manage critically ill patients is essential for selecting antibiotic dosing regimens. AUC/MIC was the main pharmacodynamic factor for tigecycline [23]. However, calculating AUC usually requires multiple consecutive samples, which is difficult to achieve in clinical ICU patients. Hence, therapeutic drug monitoring (TDM) is necessary to evaluate the clinical efficacy of tigecycline. In ICU patients, steady-state blood drug concentration monitoring plays a crucial role due to the possibility of one-time sampling. TDM studies of tigecycline use a standard dose, and there are no studies on high doses [24]. Our study found that the drug concentration of tigecycline in HD group patients was significantly higher than in the SD group. It has been the first time reported that the blood concentration of high-dose tigecycline has significantly increased. However, there was no significant association between the plasma concentration of tigecycline and clinical efficacy in our study. These results were also consistent with another study [13].

Recent studies have shown that a combination of the Acute Physiology and Chronic Health Evaluation II (APACHE II) score ≥ 24 and AUC0–12 h × V/MIC ≥ 100 (where V is the apparent distribution volume of the central compartment) is also closely related to the clinical efficacy of tigecycline [25]. Our research found that APACHE II also affected drug efficacy, consistent with previous studies’ results.

In terms of mortality, ICU days are an influential factor. A meta-analysis suggests that high-dose use of tigecycline can reduce the mortality rate of HAP [5]. This effect contrasts with our studies, possibly due to drug combination, patient age, etc. In our research, most patients received combination treatment. In a meta-analysis study, combination treatment significantly lowered the mortality risk in treating hospital-acquired pneumonia (HAP) [26]. Combination with meropenem and tigecycline showed significantly lower in-hospital mortality [27]. Combined with polymyxins, it has a good synergistic effect in vitro [28]. However, a propensity score-matched study showed that the combination therapy was not associated with clinical outcomes, mortality, and microbiological cure [29]. A recent study indicated that the ceftazidime–avibactam-based regimen was superior to the TGC-based regimen [30]. Ceftazidime–avibactam was limited in some hospitals in China, so tigecycline was still a treatment option.

One study indicated that MIC ≤ 2 mg/L was associated with tigecycline’s clinical efficacy in treating hospital-acquired pneumonia caused by MDR *A. baumannii* [31]. Our study showed no significant difference in clinical efficacy between the two groups in patients with MIC = 2 mg/l. This is not consistent with Zhou’s study. This may be why patients with MIC ≤ 2 mg/l were the main population in this study.

The resistance phenotype of CRE can be classified into two main sub-groups: non-carbapenemase-producing and carbapenemase-producing [32]. The application of whole genome sequencing in identifying antimicrobial-resistant genes has demonstrated guiding treatment decisions [33]. A recent study indicated that antibiotic resistance genes (*aac3iia*) and virulence genes (AREO-*iutA*, Capsule-*wzc*) were independent mortality risk factors for patients with carbapenemase-producing K. pneumoniae infections [34].

Studies have shown that albumin also affects the clinical efficacy of tigecycline. For every 10.3 g/L increase in the albumin, the odds of microbiological success increased 8–21 times compared to the albumin ≤ 26.00 g/L [35]. This observation may be due to the high protein-binding rate of tigecycline, which may increase the free plasma concentration and be further associated with clinical efficacy. Therefore, during tigecycline treatment, if the albumin value is below the normal limit, the albumin can be supplemented appropriately to obtain a more significant curative effect. Due to the small number of patients in our study and most patients with an albumin value of ≥ 26.00 g/L, the correlation between the albumin and PK parameters is not analyzed.

Literature research found that the adverse reactions of tigecycline were diarrhea, coagulation abnormalities, and thrombocytopenia in a real-world study [36]. Whether TDM monitoring can prevent adverse reactions is still unclear. A study reported that blood tigecycline concentration was related to hepatotoxicity [13]. Our study found no significant difference in ALT, AST, ALP, and Total bilirubin before or after tigecycline treatment in both groups. However, there was a significant decrease in fibrinogen levels in the HD group but no significant changes in APTT and PT. A retrospective study of 55 cases found that albumin level and weight-adjusted tigecycline dosage affected PT and APTT prolongation [37]. However, this study did not indicate whether patients used large doses of tigecycline. Retrospective studies suggested that large doses of tigecycline lead to fibrinogen decline [38]. Besides age, our study indicated that tigecycline treatment duration may also lead to decreased fibrinogen levels, which has not been reported in other studies. Lastly, our study found that the trough concentration of tigecycline in the blood did not predict the level of fibrinogen decline. Literature reported that Fibrinogen falls to critically low levels (< 1.0 g/L) during major hemorrhage was clinically significant [39]. However, early detection of fibrinogen decline can reduce the risk of bleeding in the ICU, and a large sample size is still needed to confirm it.

The exact mechanism by which tigecycline causes a decline in fibrinogen levels is not fully understood. It is believed that tigecycline is supposed to inhibit interleukin six (IL-6) expression, which usually stimulates gene expression and increases fibrinogen levels [40]. Recently, a study found that inhibiting mitochondrial DNA translation was a potential molecular mechanism [41]. A study showed that MDR* A. baumannii* strains can bind fibrinogen through two domain tip adhesins, Abp1D and Abp2D [42]. Plasminogen bound to CipA, which mediates complement resistance of *A. baumannii,* could be activated into plasmin to degrade fibrinogen [43]. This may explain why there were no differences between APTT and PT. Several methods have been proposed for preventing or minimizing fibrinogen decline. These include monitoring fibrinogen levels before and during treatment with tigecycline, adjusting doses based on patient characteristics such as age and underlying medical conditions, and avoiding concomitant use of other medications known to affect clotting. Additionally, it has been suggested that prophylactic administration of vitamin K may benefit some patients receiving long-term tigecycline therapy due to its role in clotting factor synthesis.

We found some limitations in our study due to i) the lack of pharmacokinetic/pharmacodynamic (PK/PD) data on tigecycline, which could lead to suboptimal dosing regimens or adverse drug effects; ii) the blood concentration of tigecycline was not the concentration of free drug. A further analysis of albumin’s effect on clinical effectiveness is not conducted.

## Conclusion

Our study found that patients receiving high-dose tigecycline exhibited significantly higher blood concentrations than those receiving low doses. The two groups had no significant differences in mortality, clinical efficacy, and bacterial eradication rates. High amounts of tigecycline were found to reduce the level of fibrinogen. Nevertheless, additional research is needed to understand the mechanism behind this reduction better and identify potential preventive measures for patients experiencing this complication during tigecycline therapy. Furthermore, further investigation is required to evaluate the efficacy of prophylactic administration of vitamin K in preventing this complication during tigecycline treatment. Based on our findings, we recommend monitoring fibrinogen levels in patients who receive high doses of tigecycline.

### Supplementary Information

**Additional file 1: Supplementary Figure S1.** Flowchart of patient enrollment. **Supplementary Figure S2.** Violin plot of the blood concentration distribution of tigecycline in the two groups. **Supplementary Table 1.** Clinical outcomes and bacterial clearance rates between the two groups for MIC = 2 mg/L.

## Data Availability

The datasets are not publicly available due to privacy or ethical restrictions but are available from the corresponding author on reasonable request.
