# Supplementary material for: Clinical efficacy and safety of tigecycline based on therapeutic drug monitoring for carbapenem-resistant Gram-negative bacterium pneumonia in intensive care units
Source: BMC Infect Dis. 2023 Nov 27;23:830. doi: 10.1186/s12879-023-08815-7 (PMC10680299; doi:10.1186/s12879-023-08815-7)
Supplement: Supplementary file 1 — Additional file 1: Supplementary Figure S1. Flowchart of patient enrollment. Supplementary Figure S2. Violin plot of the blood concentration distribution of tigecycline in the two groups. Supplementary Table 1. Clinical outcomes and bacterial clearance rates between the two groups for MIC = 2 mg/L. [file 12879_2023_8815_MOESM1_ESM.docx]

Age≥18 years at ICU from December 2020 to October 2021

N=421

Analysis study

N=45

Other site of infection (30)

kidney failure (9)

dialysis (10)

N=49

High Dose group(HD)

N=13

Standard Dose(SD) group

N=32

Acinetobacter baumannii OR Klebsiella pneumoniae with tigecycline

N=139

Supplementary Figure S1: Flowchart of patient enrollment


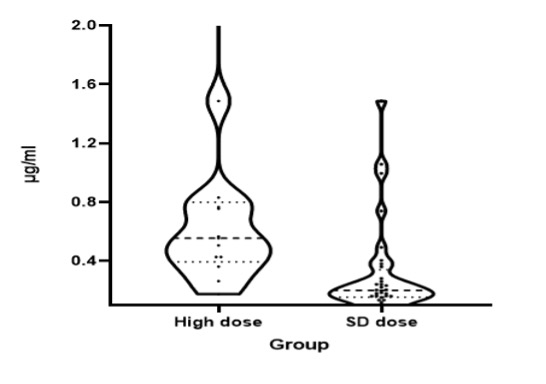


High dose means HD group; SD dose means SD group; (Confidence interval)

Supplementary Figure S2：Violin plot of the blood concentration distribution of tigecycline in the two groups

Supplementary Table 1 :clinical outcomes and bacterial clearance rates between the two groups for MIC = 2 mg/L

| Outcome | HD group (N =10) | SD group(N=23) | p value |
| --- | --- | --- | --- |
| Bacterial eradication | 3 | 7 | 0.980 |
| Mortality | 3 | 5 | 0.611 |
| Clinical efficacy | 7 | 13 | 0.466 |
